# Living with Retinitis Pigmentosa in Türkiye: Diagnosis, Independence, and Access to Care

**DOI:** 10.3390/healthcare14050593

**Published:** 2026-02-27

**Authors:** Nurcan Gürsoy, Ersan Gürsoy

**Affiliations:** 1 Department of Ophthalmology, Mengücek Gazi Training and Research Hospital, Erzincan 24100, Turkey; nurcansenturkgursoy@gmail.com; 2 Department of Family Medicine, Erzincan Binali Yıldırım University, Faculty of Medicine, Erzincan 24100, Turkey

**Keywords:** health services accessibility, personal narratives, psychological adaptation, qualitative research, retinitis pigmentosa, social participation

## Abstract

**Background:** Retinitis pigmentosa (RP) is a progressive inherited retinal dystrophy that affects daily functioning, psychological well-being, and social participation. Although quantitative research describes disease burden, less is known about how individuals experience progressive vision loss in everyday life and within healthcare and social contexts. **Methods:** This qualitative study used semi-structured face-to-face interviews with adults diagnosed with RP. Purposive sampling was applied to ensure variation in demographic and clinical characteristics. Interviews were conducted in a tertiary ophthalmology clinic in Erzincan, Türkiye, between June and October 2025. Audio recordings were transcribed verbatim and analyzed using Braun and Clarke’s reflexive thematic analysis. Reporting followed the Consolidated Criteria for Reporting Qualitative Research (COREQ) checklist. **Results:** Sixteen participants (P1–P16) were included. Five themes were identified: (1) making sense of the illness and the diagnostic journey; (2) functional loss and the negotiation of independence; (3) psychological adaptation and identity reconstruction; (4) social relationships and social encounters; and (5) interaction with systems and the environment—accessibility and healthcare. Participants described early symptom normalization, delayed diagnostic pathways, and uncertainty persisting after diagnosis. Independence was shaped by safety concerns, environmental barriers, and reliance on support. Psychological adjustment fluctuated between fear of progression and efforts to sustain resilience. Social participation was influenced by support networks, concerns about being a burden, and stigma linked to invisible disability. **Conclusions:** Living with RP extends beyond visual impairment; building on prior qualitative work, our findings contextualize these experiences in Türkiye, highlighting how accessibility gaps, bureaucratic encounters in public institutions, and cost barriers within healthcare and public services can shape uncertainty, independence, and social participation.

## 1. Introduction

Retinitis pigmentosa (RP) is a progressive group of disorders within the spectrum of inherited retinal dystrophies, characterized by photoreceptor degeneration [1,2,3]. Clinically, it is commonly marked by night blindness (nyctalopia) and functional impairment resulting from gradual constriction of the peripheral visual field over time [1,4]. The progressive nature of the disease is not limited to a decline in visual function; it can also have substantial impacts on individuals’ daily activities, independent mobility, and social participation [4,5].

RP is associated with reduced quality of life and clinically relevant anxiety/depression, and population-based evidence suggests an elevated risk of depressive disorders among individuals with RP [6,7,8,9]. These findings indicate that follow-up and care should extend beyond visual function parameters to address psychosocial needs [1,10].

Nevertheless, although quantitative studies can document disease burden, they may be limited in explaining how individuals experience progressive vision loss, cope with uncertainty, negotiate independence, and interact with their social and institutional environments. Qualitative syntheses and primary studies consistently describe themes such as diagnostic delay, uncertainty, loss of independence, stigma, and psychosocial distress, and emphasize information needs, emotional regulation, and social support as central to living with RP [11,12]. More recent qualitative work has also examined patient experience to characterize functional impacts and inform outcome measurement, but typically with less emphasis on healthcare/public-service mechanisms shaping everyday life [13,14,15]. However, prior qualitative research has less often examined the institutional mechanisms through which these experiences are shaped—such as referral pathways, administrative procedures, accessibility accommodations, and cost constraints—particularly within the healthcare and public-service context of Türkiye.

In this context, this study aims to examine the lived experience of adults with RP in Türkiye, with specific attention to how navigating healthcare and public-service systems shapes the diagnostic journey, psychosocial adaptation, independence, and social participation.

## 2. Materials and Methods

### 2.1. Study Design

This study was designed using a qualitative research approach and employed semi-structured individual interviews to explore in depth the quality of life, coping processes, and social experiences of adults living with RP. Reporting was guided by the Consolidated Criteria for Reporting Qualitative Research (COREQ) checklist [16].

### 2.2. Setting and Period

The study was conducted in Erzincan, Türkiye, in the ophthalmology clinic of a tertiary-level hospital. Erzincan is a provincial setting, and the tertiary clinic provides specialist ophthalmology care for the city and surrounding districts. Data were collected between June and October 2025. Interviews were conducted face-to-face in a private setting to ensure participants’ confidentiality.

### 2.3. Participants and Sampling

Participants were recruited using purposive sampling. The sampling strategy aimed to ensure variation in age, sex, duration since diagnosis, level of vision loss, and social/occupational status. Adults aged ≥ 18 years who had been followed for RP for at least one year were eligible. Participants with major comorbid ocular or systemic conditions likely to independently affect vision or functioning (e.g., advanced cataract, glaucoma, diabetic retinopathy, neurologic disease) were excluded to minimize confounding of RP-related experiences. Individuals with severe cognitive impairment/acute psychiatric conditions likely to substantially hinder the interview process, or with severe clinical conditions preventing completion of the interview, were excluded.

Sample size was determined according to the principle of data saturation; following each interview, transcripts were reviewed and preliminary codes were noted in a running log. Saturation was considered achieved when consecutive interviews yielded no new codes or thematic directions and served only to enrich existing themes with additional examples/quotations; recruitment was then discontinued. A total of 16 participants were included and were coded as P1, P2, etc., in the report.

### 2.4. Recruitment Procedures

Ten participants were recruited among patients attending the outpatient clinic and were informed about the study during their visit. The remaining six participants were identified through outpatient clinic records; they were approached and informed using the same procedure, and those who agreed were enrolled. Four patients who were contacted by telephone declined participation. Informed consent was obtained from all participants.

### 2.5. Data Collection Instrument and Interview Procedures

Data were collected using a semi-structured interview guide consisting of 12 questions, developed by the research team based on the literature and study objectives. The guide covered the diagnostic journey, functional loss in daily life and negotiation of independence, psychological adaptation/identity, social relationships and social encounters, and accessibility and healthcare experiences (available at https://doi.org/10.5281/zenodo.18317783).

Interviews lasted a median of 32 min (range: 25–40 min). Given participants’ visual impairment and potential fatigue, interviews were intentionally kept focused and flexible, with probing questions used to elicit depth, and data collection continued until saturation. No repeat interviews were conducted. Transcripts were not returned to participants for comment, and formal member checking was not performed. With participants’ permission, interviews were audio-recorded, and field notes were taken when deemed necessary. Interviews were further explored using probing questions.

To aid interpretability, visual acuity was summarized using the most recent clinical record at the time of interview (decimal VA converted to logMAR), with broad categories noted where applicable (CF/LP/NLP), and symptom/diagnosis duration was reported in Table 1 as contextual markers across the disease spectrum; these descriptors were used to support interpretation and ensure representation across the range of vision loss, but we did not conduct severity-stratified thematic comparisons.

### 2.6. Reflexivity

All interviews were conducted by the same researcher, an ophthalmologist with seven years of clinical experience (NG, female, 36 years). Some participants were current or former patients of the interviewing physician (P1, P4, P7, P10, P11, P12), whereas no such clinical relationship existed with the remaining participants. To mitigate potential effects on data generation, prior to the interviews it was emphasized that the interviewer was acting in the role of a researcher independent of clinical care, that participation was voluntary, and that participation/non-participation and any responses would not affect healthcare services. A non-judgmental interview style and neutral probes were used, and participants were explicitly invited to share both positive and negative accounts, including criticism of healthcare providers/encounters, to reduce social desirability. Reflexive notes were recorded after each interview and discussed during analysis with the second researcher to interrogate assumptions related to the interviewer’s dual role. These notes were used as analytic memos to inform coding decisions, document shifts in interpretation, and guide reflexive team discussions when refining theme boundaries and selecting illustrative quotations.

This study was informed by a contextualist epistemological stance, recognizing that accounts of living with RP are shaped by social, cultural, and institutional contexts. Interviews were treated as a co-produced site of meaning-making rather than an extraction of objective “facts”; accordingly, the interview guide prioritized open-ended questions and probing to explore how participants interpreted experiences and navigated care and public services. Consistent with this stance, analysis used reflexive thematic analysis to develop interpretive themes through iterative engagement with the dataset, analytic memoing, and reflexive team discussion.

### 2.7. Data Processing and Analysis

Audio recordings were transcribed verbatim shortly after each interview. Identifying information was removed to anonymize the transcripts. Data were analyzed using Braun and Clarke’s reflexive thematic analysis approach with iterative theme development moving beyond semantic description to interpretative links to communication practices, cultural norms, and system constraints [17]. We adopted a contextualist approach, treating themes as interpretive patterns developed through sustained engagement with the dataset.

Coding and data management were undertaken using NVivo 12 (Lumivero, Denver, CO, USA) software. In the initial phase, the research team repeatedly read the transcripts to develop preliminary codes, which were then organized in NVivo to create a working codebook including code definitions and exemplar quotations. During coding, two researchers independently coded a subset of transcripts; differences in coding were discussed in analytic meetings to refine code definitions and the evolving coding framework and to strengthen interpretive coherence, rather than to quantify or maximize inter-coder agreement. Following completion of coding for the full dataset, codes and candidate themes were reviewed in team meetings; thematic coherence and boundaries between themes were examined, and the thematic structure was refined through team discussions focusing on coherence, distinctiveness, and fit with the dataset. Throughout the analytic process, key decisions and revisions were documented as an audit trail, and the original transcript context was revisited when necessary to ensure interpretations remained grounded in the data.

### 2.8. Trustworthiness

To enhance rigor, methodological strategies were applied in line with the principles of credibility, transferability, dependability, and confirmability. Credibility was supported through prolonged engagement with the data, iterative coding, and team discussions to refine codes and themes. A subset of transcripts was independently coded by two researchers, and differences in coding were discussed in analytic meetings to refine the evolving coding framework and strengthen interpretive coherence. An audit trail was maintained to document key analytic decisions, revisions, and theme development. We used analyst triangulation by involving a second researcher as a critical discussion partner during coding and theme refinement to challenge assumptions and strengthen credibility. Transferability was facilitated by purposive sampling with maximum variation and thick description of the setting and participants.

### 2.9. Ethical Approval and Confidentiality

Ethical approval was obtained from the Erzincan Binali Yıldırım University Non-Interventional Clinical Research Ethics Committee (Meeting No: 09; Decision No: 2025-09/04; Date: 15 May 2025). Written informed consent was obtained from all participants. Participant identities were protected through coding, and identifying details were removed from transcripts and reports. Audio files and transcripts were stored in encrypted digital files accessible only to the research team.

## 3. Results

A total of 16 adults with retinitis pigmentosa (RP) participated in the study. Participants were aged 23–69 years, and symptom duration ranged from 15 to 57 years, representing long and heterogeneous illness trajectories. To support the interpretability of narratives across the disease spectrum, the basic clinical context was summarized using visual acuity indicators; reported vision ranged from logMAR 0.5 to 3.0, including participants with counting fingers (CF) and light perception/no light perception (LP/NLP) levels (Table 1). The themes presented below capture participants’ experiences across the diagnostic journey, psychosocial adaptation, independence, and social participation within this range of clinical severity.

### 3.1. Theme 1: Making Sense of the Illness and the Diagnostic Journey

Participants’ accounts indicate that the process of making sense of the illness typically begins with early, subtle functional impairments that are initially “normalized.” These first signs sometimes become salient in school settings: *“During middle school, I stopped noticing the writing on the board; I went to the doctor, and they gave me glasses.”* (P1). Similarly, early recognition of reading and writing difficulties in childhood was emphasized: *“My complaints started in primary school… I began to struggle when writing, and I could read by holding books close to my eyes.”* (P2). For some participants, “recognition” was sharpened by a more dramatic experience; in particular, not being able to see in the dark was linked to a risky event in everyday life: *“…I was playing in the evening; because I couldn’t see, I fell into the tandoor pit (crying). That’s when I realized I couldn’t see in the dark.”* (P8). In addition, work-related recognition in adulthood was also observed: *“I was a driver; I started having difficulty driving at night…”* (P10). In some accounts, however, early symptoms could lead to delayed meaning-making because they were assumed to be “normal for everyone”: *“…I had the complaint of low night vision for a very long time, but I thought everyone was like that…”* (P12).

On the path to diagnosis, participants described not only “seeking doctors”, but also a process involving delays, incorrect/incomplete explanations, and constraints in access. For instance, despite early consultation, initial explanations could remain within a frame that “simplified” symptoms, thereby prolonging diagnostic uncertainty: *“…they said it was vitamin deficiency, but my complaints did not go away…”* (P2). Likewise, some participants explicitly stated that access to diagnosis was delayed due to socio-practical conditions: *“I couldn’t go to the doctor right away; I could go only about five years later.”* (P14)./”*I couldn’t go to the doctor for a long time because there was no one to take me.”* (P5).

When a diagnosis was established, it often remained in participants’ memories as a “label”, both in its medical name and in colloquial language. In how the diagnosis was communicated, an emphasis on “progression/incurability” became apparent; for some, this was considered together with the possibility of familial occurrence: *“…she told me that the definitive diagnosis was retinitis pigmentosa and that this disease would not go away, and that it could also be present in my siblings.”* (P2). In addition, the expression “night blindness” recurred in narratives as the way the diagnosis had entered everyday language: *“…the doctor told me I had night blindness.”* (P10)/*”…they said you also have night blindness.”* (P12). Some participants recalled diagnosis being delivered with the phrase ‘there is nothing to be done’; beyond conveying prognosis, this closure-style communication can foreclose questions about coping, rehabilitation, and follow-up and, in time-pressured/resource-constrained services, may intensify distress and disengagement: *‘…they diagnosed retinitis pigmentosa and said there is nothing to be done.*’ (P16).

Reactions at the time of diagnosis were not uniform; a clear contrast was observed in the data. One group of participants reported experiencing “emotional freezing/inability to react” due to lack of knowledge: *“After I received the diagnosis, I didn’t react much because… I didn’t really have much information about retinitis pigmentosa.”* (P1). In contrast, some made sense of the diagnosis through a religious/fate-based language; this language could be accompanied by both acceptance and withdrawal: *“When I first learned it, I said it is what God wills, and then I stopped going to doctors…”* (P2). At the other end, the diagnosis was expressed as intense devastation and withdrawal: *“…she said there was no cure; I almost lost my mind. I shut myself in my room and cried…”* (P14). Nevertheless, within the same theme there was also a line of “active seeking despite sadness”: *“…I was very upset, but I didn’t let it go; I went to many doctors.”* (P15).

Finally, receiving a diagnosis did not always end uncertainty; some participants emphasized that, in the post-diagnosis period, needs for information and explanatory communication were not met: *“I can’t benefit adequately from health services; I can’t get detailed answers to my questions; it feels like they always brush me off…”* (P15). This finding suggests that the diagnostic journey does not end with the “label”, and that the search for information to make sense of and manage the illness continues after diagnosis; therefore, diagnosis may become not only a clinical outcome but also the beginning of an ongoing meaning-making process.

### 3.2. Theme 2: Functional Loss in Daily Life and the Negotiation of Independence

In participants’ narratives, vision loss is described not merely as a sensory deficit at the level of “low vision”, but as an experience that makes the capacity to use space, sustain daily tasks, and move safely a matter of ongoing negotiation. This negotiation is shaped, on the one hand, by the narrowing of independence (restricted outdoor mobility and reliance on support indoors; compensatory strategies such as home organization, tactile cues, and cane training). A salient contrast in the data is that while some participants increasingly “withdraw into the home”, others maintain contact with the outside world—albeit in a limited way—through assistive devices and strategies.

The restriction of mobility due to vision loss is most clearly visible in narratives of being “unable to go out alone”: *“For ten years I haven’t been able to go out on my own; I can’t move independently.”* (P16). This limitation is not only “not being able to go out”, but is also experienced as spatial safety being confined to the home: *“I memorized the inside of the house, but outside the house everything is over; I would get lost immediately.”* (P16). Similarly, independent mobility outdoors became a basic threshold for some: *“I can’t go to the bazaar on my own; I can’t cross the street; I struggle a lot.”* (P4).

Functional loss also weakens independence in domains such as household tasks and technology use. Participants emphasized that even routine home activities could not be maintained alone: *“Even when cooking at home, I can’t do it alone without someone’s help.”* (P16). This was also reflected in the use of communication/media devices: *“I can’t use the television or the phone; I only listen to the sound of the TV.”* (P16). In addition, loss of independence was experienced not only as functional but also as an emotional burden: *“…at home my spouse does everything too; I can’t even find a glass… this feeling of dependence makes me very sad…”* (P4).

Participants reported developing safety strategies to preserve independence, although these strategies often operated with a “risk management” logic. Navigation indoors was supported by tactile cues: *“When moving from one room to another, I pass by touching the walls.”* (P16). Outdoors, experiences of collision/falling emerged as factors that directly constrain independence: *“…because I couldn’t see the car, I hit my hand on it; many times I fell and hit things…”* (P16)/*”Shopkeepers put obstacles on the road; I trip and fall.”* (P11). These accounts suggest that independence is often shaped less by “desire” than by environmental hazards and accessibility conditions. From a social model of disability perspective, ‘dependence’ here is not only an individual limitation; it is produced where low vision meets an environment that is unsafe or hard to navigate (e.g., obstacles, unsafe crossings, limited wayfinding). In WHO’s ICF terms, these environmental barriers act as contextual constraints that magnify activity limitations—so participants’ ‘risk-management’ becomes necessary day-to-day work rather than a personal preference.

Nevertheless, the data also include a contrasting line in which assistive devices (especially a cane) play a more “enabling” role in rebuilding independence. One participant emphasized that cane training facilitates mobility outdoors: *“If I go out, I go out with a cane; I received cane training, and I can move comfortably outside with it.”* (P11). Similarly, establishing a “fixed order” at home and tactile orientation appears as another strategy that enables partial independence: *“…I’ve fixed the place of the items; I never change them… I can go by holding on to the wall.”* (P3).

Overall, Theme 2 shows that vision loss reorganizes daily life through the boundary between indoor and outdoor space; independence, situated between “total loss” and “full independence”, emerges as a domain continuously negotiated through the interaction of individual strategies, assistive devices, and environmental conditions.

### 3.3. Theme 3: Psychological Adaptation and the Reconstruction of Identity

In participants’ accounts, psychological adaptation appears not as a “one-time acceptance” but as a process that fluctuates between feelings of loss, fear/anxiety, shifts between hope and hopelessness, and the reconstruction of self-perception over time. Some participants described diagnosis and progression together with profound emotional collapse and the constriction of their life space: *“…she said there was no cure; when I heard that, I almost lost my mind. I shut myself in my room and cried and cried; I was very upset.”* (P14). This collapse intensifies the experience of self through a “darkness” metaphor as time progresses: *“…right now I can’t see at all; it’s as if I’m in a dark dungeon.”* (P14).

Alongside this line of emotional vulnerability, future anxiety and fear of becoming dependent were highly salient in the data. Anxiety is sometimes reduced directly to bodily dependence and sometimes articulated intertwined with spirituality: *“I have worries about the future; I cry every night… My God, don’t let me fall into someone’s hands.”* (P14). This withdrawal and the feeling of “four walls” also indicate the persistence of psychological burden: *“…I’m between four walls; I can’t go out… I struggle a lot.”* (P14). Similarly, another participant expressed fear about progression more directly: *“I have a lot of worries about the future… my condition is getting worse; I’m very afraid.”* (P15). There are also examples where anxiety is framed as “not being able to manage oneself”: *“My worry about the future is if I can’t manage myself… I’m afraid of what would happen.”* (P3). From a healthcare communication perspective, these accounts suggest that uncertainty often persists after diagnosis—especially when people receive limited practical guidance beyond prognosis. In disability theory terms, what threatens identity here is not only vision loss itself but the anticipated loss of agency and social role, which may become sharper when formal supports are limited and care shifts to family.

However, an important contrast in Theme 3 emerges through “reframing” and an identity of resilience/self-efficacy. Some participants arrive at a kind of accommodation after intense affect by adopting a “there is nothing to be done” frame: *“I cried and suffered too, but I saw there was nothing to be done, so I sat down in my place.”* (P14). This accommodation may continue alongside thoughts that oscillate between hope and hopelessness: *“…I think maybe they can find a solution by putting a lens in the brain, but then I think there is no cure, and I fall into hopelessness.”* (P14).

On the other hand, in some narratives, identity reconstruction is shaped by a strong self-efficacy discourse organized around “not being dependent on anyone”: *“…I always did my own work and tried not to be dependent on anyone… I wasn’t dependent on anyone.”* (P3). The same line is reinforced with an emphasis on continuity: *“…I never fell into hopelessness because of my disability… I wasn’t dependent on anyone, and I didn’t make my children dependent either.”* (P3). For some participants, adaptation is framed not as “being completely well”, but as sustaining daily life by managing negative affect: *“…I tried to continue my work without falling into unhappiness.”* (P14).

Overall, Theme 3 shows that psychological adaptation to vision loss makes loss and vulnerability visible while also generating different pathways for reconstructing identity through spirituality, acceptance, and self-efficacy; in some participants, these pathways are not experienced without tension but rather coexist in a fluctuating manner over time.

### 3.4. Theme 4: Social Relationships and Social Encounters

According to the accounts of the participants, vision loss alters social life on two distinct levels: firstly, it necessitates the restructuring of care and support networks within intimate relationships; secondly, it results in the need to justify oneself and face discrimination in public areas because of the “invisible disability.” This theme highlights the “protective” aspect of support while showing that the same support can also be emotionally challenging by reminding individuals of dependence and triggering a sense of “being a burden.” It is also evident that social encounters are not experienced as equally negative by all participants; while some narratives contain clear examples of stigma, others create contrast by stating, “I did not experience negativity.”

At the level of close relationships, support is described as a critical resource for sustaining daily life. One participant depicts how tangible this support can become: *“My environment—my family and friends—supports me… The most support I receive is from my spouse; my spouse even puts my clothes on me.”* (P11). Similarly, access to follow-up care is supported through accompaniment: *“…I received a lot of support from my family and my spouse; my spouse brought me to my check-ups.”* (P15). However, this support relationship is not always experienced as something that “feels good”; the continuity of help can sharpen the feeling of dependence: *“…at home my spouse does everything too; I can’t even find a glass… this feeling of dependence makes me very sad…”* (P4).

The idea of ‘being a burden’ functions as a moral–relational constraint on social participation, reflecting norms of reciprocity/family responsibility and becoming amplified where accessible environments and formal support are limited. For some participants, this worry turns directly into social withdrawal: *“I try not to leave the house because if I go to someone, I’m afraid I will inconvenience that person.”* (P11). Another dimension accompanying this is that using assistive devices (e.g., a cane) can be intertwined with “shame” and concern about drawing attention: *“…I can’t bring myself to walk with a cane; I’m ashamed; I feel as if everyone is looking at me…”* (P10).

Social encounters in public spaces are particularly shaped by experiences of being misjudged and facing prejudice related to “invisible disabilities.” For some participants, this emerges as a clear stigma narrative: *“…when I tap my disability card on buses, there are people who judge me.”* (P11). In some narratives, prejudice produces a “hesitancy” that reduces participation in social environments: *“Society behaves with prejudice toward people with low vision like me… you hesitate to enter a place, you can’t make friends, and you feel excluded.”* (P5). Stigma can even be experienced within the family and may turn into concealment framed by “shame/fault”: *“My family was even ashamed of me… they saw my low vision as a defect and hid it from everyone…”* (P5).

In contrast, not all participants’ social encounters are negative; some clearly state they felt included: “*I don’t think I am excluded by society; no one says anything bad*.” (P15). This contrast suggests that social experience does not progress along a single line but can be shaped differently depending on factors such as the degree of visibility, the intensity of need for help, the attitudes of the surrounding environment, and how individuals position themselves in social settings.

### 3.5. Theme 5: Interaction with Systems and the Environment—Accessibility and Healthcare

In participants’ accounts, interaction with systems is not limited to seeking healthcare; it also includes everyday bureaucratic experiences such as handling procedures in public institutions, service design that does not accommodate disability, and the constant need to explain oneself due to invisible disability. These experiences indicate that vision loss can shift from being a “personal condition” to becoming a field of difficulty co-produced by institutional arrangements and environmental conditions. For some participants, the main problem is the absence of facilitation and the “wait your turn like everyone else” approach: *“When I go to crowded institutions, there is no special practice for us; they say wait your turn, they show no understanding.”* (P11). For others, institutional contact requires repeated explanations because functional loss becomes visible during processes such as signing: *“…especially when signing, I struggle a lot; I always have to explain that I have low vision.”* (P9).

Engagement with healthcare services also produces a heterogeneous experience among participants. On the one hand, there are narratives suggesting they can benefit from the healthcare system (*“I think I can benefit adequately from health services…”* P11). On the other hand, experiencing the “there is no cure” frame as a communication style strengthens feelings of not being taken seriously and being dismissed for some: *“…I think they don’t take my illness seriously; they say there is no cure, don’t come anymore.”* (P9). This indicates that the way clinical information is communicated can have not only medical but also psychosocial consequences (hopelessness/withdrawal).

Another salient line in Theme 5 concerns treatment-seeking shaped by cost and an “economy of hope”. Participants report exploring treatment options, but also that economic barriers can limit possibilities: *“They said there is stem cell treatment, but it was paid; we couldn’t afford it, we couldn’t do it.”* (P11). In some narratives, repeated consultations, spending, and disappointment coexist: *“…I searched for a remedy; hospitals took a lot of my money, but I couldn’t find a remedy.”* (P9). The chronic nature of this search produces a dual outcome—sustaining hope while also becoming worn down: *“I keep going to the doctor to see if there is something new; I am looking for hope…”* (P9). Continuing to try interventions on the basis of “hope” is also evident: *“…I had PRP, but I didn’t see much benefit… but we tried it thinking it’s still a hope.”* (P9).

Overall, Theme 5 indicates two critical points in participants’ interaction with systems: (i) institutional-level inadequacy of accessibility (procedures, signing, crowded institutions, lack of facilitation) and (ii) how communication/guidance and economic access in healthcare meaningfully shape life after diagnosis. These findings suggest that uncertainty can persist even “after diagnosis”, and that the responses offered by the system and the conditions of access directly influence the experience of living with the illness.

## 4. Discussion

In this qualitative study, we examined the diagnostic process, daily functioning, psychosocial adjustment, and social participation of individuals living with RP in Türkiye. Overall, RP is not limited to progressive visual loss; rather, it represents a long-term lived experience that reshapes uncertainty management, the negotiation of independence, identity, and social participation. While our themes resonate with prior qualitative syntheses [11,12], a key contribution of this study is comparative contextualization: the existing meta-synthesis drew on studies from Australia, Brazil, Ireland, the Netherlands, the Republic of Korea, the United Kingdom, and the USA [11], whereas qualitative evidence from Türkiye has been relatively limited. In this setting, participants described how accessibility gaps, bureaucratic procedures in public institutions, and cost barriers co-shape uncertainty, independence, and social participation. Such “bureaucratic encounters” are consistent with the concept of administrative burden in citizen–state interactions (learning, psychological, and compliance costs) [18], and align with reported structural barriers to assistive-technology access and rehabilitation supports among people with visual impairments in Türkiye [19].

One of the key observations in our study is that, for many participants, the diagnostic process was not experienced as a “final answer”, but often as the beginning of a new period marked by uncertainty. The normalization of symptoms before diagnosis, delayed help-seeking, and anxiety accompanying the diagnostic period were notable in participants’ narratives. This aligns with quantitative evidence indicating that the psychological burden of RP can be clinically meaningful [7,20,21,22]. In particular, studies in young adults with RP have reported associations between visual function and quality of life, and have highlighted notable levels of mental health scores suggestive of anxiety and depression [6]. In addition, large-scale population-based cohort data show that the risk of developing depressive disorders increases following an RP diagnosis [7]. Taken together, these findings support the view that RP follow-up should not be limited to visual function measurements but should be maintained through a holistic approach that also addresses psychosocial impact [1,10].

A second important finding is that vision loss transforms independence in daily life from a “stable attribute” into a condition that is reconstructed and negotiated over time. Participants’ accounts demonstrated that loss of independence becomes tangible through experiences such as a restricted life space, safety concerns in outdoor environments, and the need to plan routine activities more carefully. In this respect, a purely biomedical focus on disease progression may not be sufficient; it is also important to incorporate individuals’ practical strategies and support needs aimed at maintaining functioning and social participation [11,23].

Third, our findings indicate that psychosocial adjustment does not follow a single, linear “acceptance” trajectory but is instead experienced alongside fluctuations and future-oriented anxiety. While participants attempted to reorganize their lives, they also had to manage uncertainty and the fear of progression. Syntheses of qualitative evidence have similarly shown that coping with RP is a major determinant of quality of life, and that information needs, emotional regulation, and social support play central roles in this experience [11,13,24]. This provides a strong explanatory context for why participants in our study repeatedly emphasized the need for access to information and clear guidance.

Fourth, social life emerged as a two-sided domain that may generate both support and vulnerability. While close social support facilitated everyday functioning, it could also strengthen feelings of being a “burden” in some participants and contribute to withdrawal behaviors. Moreover, the fact that RP is not always outwardly visible increased the likelihood of misunderstanding in social encounters and created a recurring need for self-explanation. Within this context, participants described having to legitimize their impairment in public settings (e.g., when requesting assistance or using disability-related services), suggesting that stigma was tied not only to disability labels but also to expectations around visibility and social roles. This pattern suggests that RP should be evaluated not only as a “medical” condition but also in relation to social participation and relational networks [4,5,25].

These findings have several clinically actionable implications: at diagnosis, structured counselling (clear explanation of prognosis, realistic yet hope-preserving communication, and a brief written information/follow-up plan) and early referral pathways to low-vision rehabilitation (low-vision services, assistive devices, and orientation-and-mobility training) should be offered; during follow-up, brief routine screening for psychological distress with referral pathways when indicated may strengthen RP care; at a system level, priority appointment/queue options, accessible signage/wayfinding, and disability-friendly administrative procedures in healthcare and public institutions may reduce avoidable barriers. Evidence on the elevated risk of depressive disorders further supports that such integrated care is a clinical requirement rather than a matter of ‘good intention’ [7].

### Limitations

By nature, qualitative findings provide in-depth interpretations within a specific context and sample. As this study was conducted in a single center and participants had a particular level of access to healthcare services, generalizability to different socioeconomic conditions or regional contexts may be limited. Because this is a single-center study from Erzincan, bureaucratic and accessibility experiences may differ across rural and metropolitan regions; therefore, transferability within Türkiye and internationally should be interpreted cautiously. In addition, the cross-sectional qualitative design does not allow for observation of how experiences change over time across different stages of RP. Nevertheless, purposive sampling and thematic analysis offered a strong narrative basis for capturing the multidimensional aspects of living with RP. Additionally, for a subset of participants, the clinician–participant relationship may have influenced disclosure through perceived power dynamics or social desirability, including reluctance to criticize healthcare providers.

## 5. Conclusions

Overall, our findings indicate that living with RP is a multidimensional experience shaped by diagnostic uncertainty, functional limitations in daily life, fluctuating psychosocial adjustment, and social encounters. Therefore, RP follow-up should not focus solely on clinical measurements; rather, there is a clear need for holistic care approaches supported by understandable information, psychosocial support, and guidance aimed at sustaining functioning. At a system level, disability-friendly administrative procedures and accessible public environments may reduce avoidable barriers following diagnosis.

## Figures and Tables

**Table 1:** Participant sociodemographic and clinical context.

ID	Age (Years)	Sex	Symptom Duration (Years)	Years Since Diagnosis	Marital Status	Children (n)	Occupation	Reported Vision *	logMAR
P1	28	M	20	3	Single	0	Farmer	0.1	1.0
P2	65	M	57	57	Married	4	Retired	CF at 1 m	2.0
P3	44	M	36	20	Married	1	Retired	CF at 2 m	1.6
P4	48	M	40	40	Married	0	Not working	LP+	2.7
P5	53	F	42	20	Single	0	Not working	LP+	2.7
P6	41	M	32	25	Married	2	Civil servant	0.2	0.7
P7	48	M	31	31	Single	0	Civil servant	0.3	0.5
P8	62	F	55	55	Married	4	Not working	LP+	2.7
P9	49	M	42	35	Single	0	Self-employed	0.1	1.0
P10	42	M	15	15	Married	2	Retired	CF at 2 m	1.6
P11	24	F	16	11	Married	1	Not reported	0.3	0.5
P12	39	F	25	10	Married	2	Not reported	0.2	0.7
P13	66	M	50	35	Married	3	Retired	CF at 5 m	1.3
P14	69	F	49	43	Married	4	Not working	NLP	3.0
P15	40	F	25	25	Married	2	Not working	0.1	1.0
P16	23	F	16	12	Married	0	Not working	CF at 1 m	2.0

* CF = counting fingers, LP = light perception, NLP = no light perception.

## Data Availability

Due to the sensitive nature of qualitative interview data and the risk of participant identification, full interview transcripts are not publicly available. De-identified excerpts supporting the findings are included in the manuscript. Additional information may be provided by the corresponding author upon reasonable request.
